# Primary Anastomosis Versus End-Ostomy in Left-Sided Colonic and Proximal Rectal Cancer Surgery in the Elderly Dutch Population: A Propensity Score Matched Analysis

**DOI:** 10.1245/s10434-021-09976-y

**Published:** 2021-04-25

**Authors:** Yu Ting van Loon, Felice N. van Erning, Huub A. Maas, Laurents P. S. Stassen, David D. E. Zimmerman

**Affiliations:** 1 Department of Surgery, Elisabeth-TweeSteden Hospital, Tilburg, The Netherlands; 2 Department of Research and Development, Netherlands Comprehensive Cancer Organisation, Utrecht, The Netherlands; 3 Department of Geriatrics, Elisabeth-TweeSteden Hospital, Tilburg, The Netherlands; 4 Department of Surgery, Maastricht University Medical Center, Maastricht, The Netherlands

## Abstract

**Background:**

Primary anastomosis (PA) in left-sided colorectal cancer (CRC) surgery in elderly patients is disputed. The aim of our study was to evaluate the differences in postoperative outcomes after left-sided CRC surgery in elderly patients in The Netherlands, comparing patients with PA and those who underwent end-ostomy (EO).

**Method:**

Patients aged ≥ 75 years with stage I–III left-sided CRC, diagnosed and surgically treated in 2015–2017 were selected from the Netherlands Cancer Registry (*n* = 3286). Postoperative outcomes, short-term (30-, 60-, and 90-day) mortality and 3-year overall and relative survival were analyzed, stratified by surgical resection with PA versus EO. Propensity score matching (PSM) and multivariable logistic regression analysis were conducted.

**Results:**

Patients with higher age, higher American Society of Anesthesiologists classification and higher tumor stage, a perforation, ileus or tumor located in the proximal rectum, and after open or converted surgery were more likely to receive EO. No difference in anastomotic leakage was seen in PA patients with or without defunctioning stoma (6.2% vs. 7.0%, *p* = 0.680). Postoperative hospital stay was longer (7.0 vs. 6.0 days, *p* < 0.0001) and more often prolonged (19% vs. 13%, *p* = 0.03) in EO patients. Sixty-day mortality (2.9% vs. 6.4%, *p* < 0.0001), 90-day mortality (3.4% vs. 7.7%, *p* < 0.0001), and crude 3-year survival (81.2% vs. 58.7%, *p* < 0.0001) were significantly higher in EO patients, remaining significant after multivariable and PSM analysis.

**Conclusion:**

There are significant differences between elderly patients after left-sided CRC surgery with PA versus EO in terms of postoperative length of stay, short-term survival, 3-year overall survival, and relative survival at disadvantage of EO patients. This information could be important for decision making regarding surgical treatment in the elderly.

**Supplementary Information:**

The online version contains supplementary material available at 10.1245/s10434-021-09976-y.

The incidence of colorectal cancer (CRC) is still increasing in Eastern Europe, Asia, and South America; however, the incidence rate and mortality is stabilizing or even declining in the US, New Zealand, Australia, and several Western European countries, including The Netherlands.^1^,^2^ Approximately 50% of patients with CRC in Europe and the US are older than 70 years,^3^ and hence elderly patients are therefore rapidly becoming the ‘new normal’ CRC surgical population and our surgical treatment should be adapted to these patients accordingly.

Few surgeons prefer the morbidity of an ileostomy over the low risk of an anastomotic leakage after right hemicolectomy. In a recent international audit, 95% of the patients after right hemicolectomy received a primary anastomosis (PA);^4^ however, the choice of an anastomosis in left-sided CRC used to be heavily disputed. The retrospective Dutch Total Mesorectal Excision trial showed a mortality rate of 57% in elderly patients, compared with 8.2% in younger patients, when there was an anastomotic leakage.^5^ Age is an important risk factor for 30-day mortality and all types of general complications, but not for anastomotic leakage.^6^ Despite this, up to 35% of Dutch elderly patients still receive an ostomy after CRC surgery.^7^ Older Dutch patients with an ostomy do not experience more ostomy-related limitations, nor a decrease in quality of life compared with their younger counterparts.^8^ Nevertheless, according to the American College of Surgeons National Surgical Quality Improvement Program (ACS NSQIP) surgical risk calculator, this population benefits from less postoperative morbidity and mortality after a PA compared with patients who were treated with an end-ostomy (EO).^9^ Unfortunately, no Dutch or European equivalent of this risk calculator is available for CRC surgery.

More information on the use and effect of ostomies can be useful in preoperative patient counseling and shared decision making. The aim of our study was to evaluate the differences in postoperative outcomes, hospital admission, short-term mortality, and 3-year overall and relative survival after left-sided colon cancer and rectal cancer surgery in elderly patients in The Netherlands, comparing the outcomes of patients after PA with those with EO.

## Methods

### Data Collection

Data from the Netherlands Cancer Registry (NCR), a population-based registry covering all newly diagnosed malignancies in The Netherlands as notified by the automated pathological archive (PALGA) and the National Registry of Hospital Discharge Diagnoses (LMR), were used. Information on patient and tumor characteristics, diagnosis, and treatment are routinely extracted from the medical records by trained administrators of the NCR. The anatomical site of the tumor is registered according to the International Classification of Diseases for Oncology (ICD-O). The UICC TNM (Union for International Cancer Control tumor-node-metastasis) classification is used for stage notification of the primary tumor, according to the edition valid at the time of diagnosis, and performance status is (re)coded according to the WHO, as described by Ma et al.^10^ Comorbidity is registered according to a modified version of the Charlson Comorbidity Index and for a subgroup only, i.e. for all patients diagnosed in 2015 and for patients from one region in The Netherlands from 2016 onwards. The collected data includes patient and tumor characteristics, American Society of Anesthesiologists (ASA) classification, performance status, comorbidity, postoperative length of stay, anastomotic leakage, and postoperative and long-term mortality. Anastomotic leakage is defined as leakage of intestinal fluids or abscess formation at the place of the anastomosis that requires either a surgical or radiological reintervention, or treatment with an endosponge, within 60 days postoperatively. As a proxy for a complicated postoperative course, a prolonged postoperative hospital admission (defined as > 14 days) was used. Patients’ vital status is obtained by annual linkage of the NCR to the Municipal Personal Records Database, which records information on the vital status of Dutch inhabitants. Follow-up on vital status was complete to 31 January 2020.

### Study Population

The present study included patients aged ≥ 75 years with stage I–III left-sided colon cancer or proximal rectal cancer, who were diagnosed in 2015–2017 and underwent surgical resection of the tumor. Left-sided colon cancer was defined as being located in the left part of the transverse colon, and the descending or sigmoid colon (C18.4–18.7). Proximal rectal cancer encompassed tumors located in the rectosigmoid (C19.9) and tumors ≥ 10 cm from the anus in the rectum (C20.9). Only patients who underwent a single surgical resection, being a transverse colon resection, left hemicolectomy, sigmoidectomy (including Hartmann’s resection), or low anterior resection were included. Furthermore, emergency resections and (extended) right hemicolectomy procedures were excluded. In case of multiple tumors per patient, only the tumor with the earliest date of diagnosis was selected. When the date of diagnosis was equal, the tumor with the most advanced stage was selected. For TNM stage, pathological stage was used. In case pathological stage was unknown or missing, clinical TNM stage was used.

Patients were categorized into two groups based on whether they received an anastomosis or an EO and, if so, which type. The first group entailed all patients with an anastomosis with or without a defunctioning ostomy and was labeled as ‘primary anastomosis’ (PA). The second group encompassed all patients who received an EO without PA and was labeled as such. Patients with an unknown type of ostomy were excluded.

### Propensity Score Matched Sample

Because of the population-based nature of the data, comparing outcomes of patients who received a PA with patients who received an EO is biased. Therefore, a subsample was created using propensity score matching (PSM), to reduce treatment assignment bias and create comparable groups. Propensity scores were determined using a logistic regression model in which the dependent variable was surgical resection with PA versus EO and the independent variables were factors potentially associated with this variable, i.e. sex, age, American Society of Anesthesiologists (ASA) classification, comorbidity, year of diagnosis, tumor location, tumor stage, differentiation grade, ileus, perforation, surgical approach, and neoadjuvant treatment. The propensity score represented the probability that a patient would receive an EO. On the basis of the propensity scores, patients who received an EO were matched 1:3 to patients who received a PA, optimizing the closeness of the matches by assigning the closest matches first. Individuals were matched on propensity scores using a caliper width equal to 0.2 of the standard deviation of the logit propensity score.

### Statistical Analyses

For both the study population and the PSM sample, differences in patient, tumor, and treatment characteristics between patients who underwent a surgical resection with PA versus EO were analyzed using Chi-square tests.

In the total study population, multivariable logistic regression analysis was conducted to assess which patient, tumor, and treatment characteristics influenced the probability of receiving an EO. Variables included in this analysis were sex, age, ASA classification, performance status, year of diagnosis, location of the tumor, tumor stage, differentiation grade, ileus, perforation, resection type, surgical approach, and neoadjuvant treatment.

Between the groups of patients who underwent a surgical resection with PA versus EO, differences in length of hospital stay, prolonged hospital admission, and postoperative 30-, 60-, and 90-day mortality were calculated using the Wilcoxon rank-sum test, Chi-square test, or Fisher’s exact test as appropriate. Multivariable logistic regressions were used to assess if an EO was associated with prolonged hospital admission and postoperative 90-day mortality after adjustment for other variables. These variables were the same patient, tumor, and treatment characteristics as listed in the logistic model above.

Within the PA group, differences in the occurrence of an anastomotic leakage and/or abscess between patients with no ostomy versus a defunctioning ostomy were assessed using the Chi-square test.

After stratification by surgical resection with PA versus EO, crude 3-year overall and relative survival rates were calculated. Relative survival is defined as the ratio of the absolute survival observed among cancer patients and the survival that would have been expected for a comparable group from the general population (same age, sex, and period). Expected survival was calculated from population life tables from The Netherlands. Multivariable Cox regression was used to evaluate the independent impact of receiving an EO versus PA on the risk of death. For the calculation of relative excess risk of death (RER), multivariable regression models with a Poisson error structure were fitted. Both multivariable models were adjusted for the aforementioned characteristics and additionally for prolonged hospital admission and adjuvant treatment. Overall survival time was defined as the time between the date of resection to the date of death or last follow-up. Relative survival was measured from 90 days after surgery to overcome the higher-risk postsurgical period.

All analyses on short-term outcomes and survival were performed for both the total study population and the PSM sample. Furthermore, analyses were also repeated for the subgroup of patients from the total study population for whom comorbidity was registered.

*P *values < 0.05 were considered statistically significant. SAS/STAT^®^ statistical software (version 9.4; SAS Institute Inc., Cary, NC, USA) was used for all analyses.

## Results

The total study population consisted of 3286 patients (male/female: 1973/1313; median age 79 years), of whom 2661 (81%) received a PA and 625 (19%) received an EO. Within the PA group, a minority of patients received a defunctioning ostomy (*n* = 227, 9%). Most patients had an ASA classification of II (54%) or III (33%). Performance status was known for 1351 (41%) patients, of whom half had a score of 0. Comorbidity was registered for 54% of patients, and a large majority (79%) had a tumor located in the left-sided colon. There were considerable differences in patient and tumor characteristics between patients who received a PA and patients who received an EO (Table 1). Defunctioning ostomies were mostly ileostomies, while almost all EO were colostomies.

**Table 1.** Patient, tumor, and treatment characteristics of the total study population (*n* = 3286) and the propensity score matched sample (*n* = 1392) according to surgical resection with primary anastomosis versus end-ostomy

	Total study population			PSM sample		
	Primary anastomosis	End-ostomy	*p* value	Primary anastomosis	End-ostomy	*p* value
*Sex*			0.229			0.66
Male	1611 (61)	362 (58)		620 (59)	202 (58)	
Female	1050 (39)	263 (42)		424 (41)	146 (42)	
*Age, years*			**< 0.0001**			0.629
75–79	1525 (57)	186 (30)		392 (38)	132 (38)	
80–84	814 (31)	242 (39)		419 (40)	131 (38)	
≥85	322 (12)	197 (31)		233 (22)	85 (24)	
*ASA classification*			**< 0.0001**			0.668
I	167 (6)	16 (3)		35 (3)	8 (2)	
II	1508 (57)	259 (41)		471 (45)	163 (47)	
III	807 (30)	291 (47)		444 (43)	144 (42)	
IV	39 (2)	26 (4)		32 (3)	8 (2)	
Unknown	140 (5)	33 (5)		62 (6)	25 (7)	
*WHO performance status*			**< 0.0001**			0.946
0	599 (22)	96 (15)		182 (17)	64 (19)	
1	424 (16)	88 (14)		138 (13)	43 (12)	
2–4	102 (4)	42 (7)		50 (5)	18 (5)	
Unknown	1536 (58)	399 (64)		674 (65)	223 (64)	
*Number of comorbidities*^*a*^			**0.0001**			0.621
0	612 (42)	96 (31)		168 (31)	62 (34)	
1	521 (36)	120 (38)		215 (39)	65 (36)	
≥2	327 (22)	99 (31)		163 (30)	55 (30)	
*Year of diagnosis*			0.172			0.921
2015	943 (35)	197 (32)		357 (34)	123 (35)	
2016	869 (33)	220 (35)		342 (33)	113 (33)	
2017	849 (32)	208 (33)		345 (33)	112 (32)	
*Location of the tumor*			**< 0.0001**			0.608
Left-sided colon	2164 (81)	420 (67)		879 (84)	297 (85)	
Proximal rectum	497 (19)	205 (33)		165 (16)	51 (15)	
*Tumor stage*			**< 0.0001**			0.619
I	725 (27)	107 (17)		207 (20)	61 (18)	
II	1018 (38)	276 (44)		462 (44)	161 (46)	
III	998 (35)	242 (39)		375 (36)	126 (36)	
*Differentiation grade*			0.572			0.894
Well/moderate	2329 (87)	549 (88)		906 (87)	299 (86)	
Poor/undifferentiated	127 (5)	34 (5)		59 (6)	20 (6)	
Unknown	205 (8)	42 (7)		79 (7)	29 (8)	
*Ileus*			**< 0.0001**			0.987
No	2441 (92)	520 (83)		899 (86)	300 (86)	
Yes	165 (6)	100 (16)		131 (13)	43 (12)	
Unknown	55 (2)	5 (1)		14 (1)	5 (2)	
*Perforation*			**< 0.0001**			0.685
No	2485 (93)	558 (89)		962 (92)	316 (90)	
Yes	57 (2)	44 (7)		38 (4)	16 (5)	
Unknown	119 (5)	23 (4)		44 (4)	16 (5)	
*Resection type*			**< 0.0001**			0.816
Transversum resection	159 (6)	17 (3)		50 (5)	13 (4)	
Left hemicolectomy	507 (19)	93 (15)		190 (18)	68 (20)	
Sigmoid resection	1275 (48)	459 (73)		649 (62)	214 (61)	
Low anterior resection	720 (27)	56 (9)		155 (15)	53 (15)	
*Surgical approach*			**<0.0001**			0.73
Laparoscopic	2035 (76)	360 (57)		657 (63)	211 (61)	
Laparoscopic converted to open	262 (10)	85 (14)		146 (14)	53 (15)	
Open	364 (14)	180 (29)		241 (23)	84 (24)	
*Location stoma*			**< 0.0001**			**< 0.0001**
Ileostomy	182 (7)	15 (2)		59 (6)	12 (3)	
Colostomy	45 (2)	610 (98)		29 (3)	336 (97)	
No stoma	2434 (91)	0 (0)		956 (91)	0 (0)	
*Neoadjuvant treatment*			**0.001**			0.685
None	2463 (93)	550 (88)		976 (93)	326 (94)	
Radiotherapy	131 (5)	47 (8)		47 (5)	13 (4)	
Chemoradiation	67 (2)	28 (4)		21 (2)	9 (2)	
*Adjuvant chemotherapy*			**< 0.0001**			0.063
No	2366 (89)	598 (96)		944 (90)	326 (94)	
Yes	295 (11)	27 (4)		100 (10)	22 (6)	

Significant values (*p* < 0.05) are given in bold

Data are expressed as *n* (%)

*PSM* propensity score matched, *ASA* American Society of Anesthesiologists

^a^Comorbidities were available for a subgroup

The PSM sample consisted of 1392 patients: 348 (57%) of the EO patients could be matched to 1044 patients in the PA group. There were no differences in patient and tumor characteristics between both groups (Table 1).

### Factors Associated with Receiving an End-Ostomy Versus Primary Anastomosis

Table 2 presents the crude percentages and adjusted odds ratios (OR) for receiving an EO. Patients with advanced age, higher ASA classification, tumor located in the proximal rectum, tumor stage III, an ileus, or a perforation were more likely to receive an EO. Furthermore, patients who underwent a sigmoid resection and patients who underwent open or converted (from laparoscopic to open) surgery also had higher odds of receiving an EO. Additionally, in a subgroup analysis of patients for whom comorbidity was known, it was found that patients with two or more comorbidities were also more likely to receive an EO than patients without comorbidity [23% vs. 14%, adjusted OR 1.76, 95% confidence interval (CI) 1.19–2.62].

**Table 2.** Crude percentages and adjusted odds ratios for receiving an end-ostomy versus primary anastomosis among the total study population

	Crude end-ostomy (%)	Adjusted OR^a^ (95% CI)
*Sex*		
Male	18	1.00 (reference)
Female	20	1.05 (0.84–1.30)
*Age, years*		
75–79	11	1.00 (reference)
80–84	23	**1.93 (1.52–2.46)**
≥85	38	**4.10 (3.11–5.39)**
*ASA classification*		
I	9	**0.52 (0.27–0.97)**
II	15	1.00 (reference)
III	27	**1.91 (1.52–2.40)**
IV	40	**3.17 (1.73–5.83)**
*Performance status*		
0	14	1.00 (reference)
1	17	0.94 (0.64–1.38)
2–4	29	1.57 (0.94–2.62)
*Year of diagnosis*		
2015	17	1.00 (reference)
2016	20	0.86 (0.67–1.12)
2017	20	**0.76 (0.59–0.99)**
*Location of the tumor*		
Left-sided colon	16	1.00 (reference)
Proximal rectum	29	**20.43 (13.27–31.47)**
*Tumor stage*		
I	13	1.00 (reference)
II	21	**1.48 (1.10–1.98)**
III	21	1.34 (0.98–1.82)
*Differentiation grade*		
Well/moderate	19	1.00 (reference)
Poor/undifferentiated	21	0.99 (0.62–1.58)
*Ileus*		
No	18%	1.00 (reference)
Yes	38%	**1.91 (1.37–2.65)**
*Perforation*		
No	18	1.00 (reference)
Yes	44	**3.07 (1.86–5.07)**
*Resection type*		
Transversum resection	10	**0.44 (0.25–0.80)**
Left hemicolectomy	16	1.00 (reference)
Sigmoid resection	26	**2.03 (1.53–2.71)**
Low anterior resection	7	**0.06 (0.04–0.11)**
*Surgical approach*		
Laparoscopic	15	1.00 (reference)
Laparoscopic with conversion to open	25	**1.87 (1.36–2.57)**
Open	33	**2.60 (1.98–3.39)**
*Neoadjuvant treatment*		
None	18	1.00 (reference)
Radiotherapy	26	0.83 (0.49–1.43)
Chemoradiation	29	1.08 (0.54–2.17)

Significant values (*p* < 0.05) are given in bold

*OR* odds ratio, *CI* confidence interval, *ASA* American Society of Anesthesiologists

^a^Adjusted for all variables listed. ‘ASA classification unknown’, ‘performance status unknown’, ‘differentiation grade unknown’, ‘ileus unknown’, and ‘perforation unknown’ were included in the analysis but results not shown

The proportion of patients receiving an EO varied considerably between hospitals: from 0 to 59% (median 18.4%, interquartile range 8.8–25.0%, calculated over 72/75 hospitals with ≥ 10 patients from the study population). There were no differences in EO between university versus non-university hospitals: 20.0% vs. 19.0% (*p* = 0.767).

### Short-Term Outcomes

The occurrence of an anastomotic leakage and/or abscess was known for 98% (*n* = 2603) of patients with PA. There was no statistically significant difference in the occurrence of an anastomotic leakage and/or abscess between patients with or without a defunctioning stoma (6.2% vs. 7.0%, *p* = 0.680).

Postoperative hospital stay was longer and more often prolonged (i.e. > 14 days) in the EO group (Table 3), both in the total study population and in the PSM sample. In multivariable analysis, patients with EO were still more likely to have a prolonged hospital admission compared with patients with PA (adjusted OR 1.61, 95% CI 1.22–2.11). Furthermore, postoperative mortality 30, 60, and 90 days after surgery was higher in patients with EO (Table 3). However, the association with 90-day mortality was no longer significant in multivariable analysis in which correction for sex, age, ASA classification, performance status, year of diagnosis, location of the tumor, tumor stage, differentiation grade, ileus, perforation, resection type, surgical approach, and neoadjuvant treatment was undertaken (adjusted OR 1.29, 95% CI 0.84–1.99) [electronic supplementary Table S1]. Subgroup analyses among patients with known comorbidity provided similar results (data not shown).

**Table 3.** Length of hospital stay, prolonged hospital admission, and postoperative mortality according to surgical resection with primary anastomosis versus end-ostomy among the total study population and the propensity score matched sample

	Total study population			PSM sample		
	Primary anastomosis	End-ostomy	*p *value	Primary anastomosis	End-ostomy	*p *value
*Length of hospital stay, days*			**< 0.0001**			**< 0.0001**
[median (IQR)]	5 (4–8)	7 (6–13)		6 (4–9)	7 (5–13)	
*Prolonged hospital admission [n (%)]*			**< 0.0001**			**< 0.0001**
Yes						
No	288 (11)	124 (20)		119 (11)	72 (21)	
Unknown	2355 (88)	494 (79)		921 (88)	273 (78)	
	18 (1)	7 (1)		4 (1)	3 (1)	
*Postoperative 30-day mortality*			**0.0004**			0.366
% Death	2.10%	4.60%		2.80%	3.70%	
*Postoperative 60-day mortality*			**< 0.0001**			**0.044**
% Death	2.90%	6.40%		3.50%	6.00%	
*Postoperative 90-day mortality*			**< 0.0001**			**0.029**
% Death	3.40%	7.70%		4.00%	6.90%	

Significant values (*p* < 0.05) are given in bold

*PSM* propensity score matched, *IQR* interquartile range

In the PSM sample, postoperative mortality 30 days after surgery was no longer statistically significant; however, mortality 60 and 90 days after surgery remained statistically significantly higher in patients with EO (Table 3). Furthermore, in univariable and multivariable analysis, patients with ASA IV, a perforation, after open surgery, and with EO had higher odds of dying within 90 days compared with patients with PA (electronic supplementary Table S1).

### Survival

**Fig. 1.**
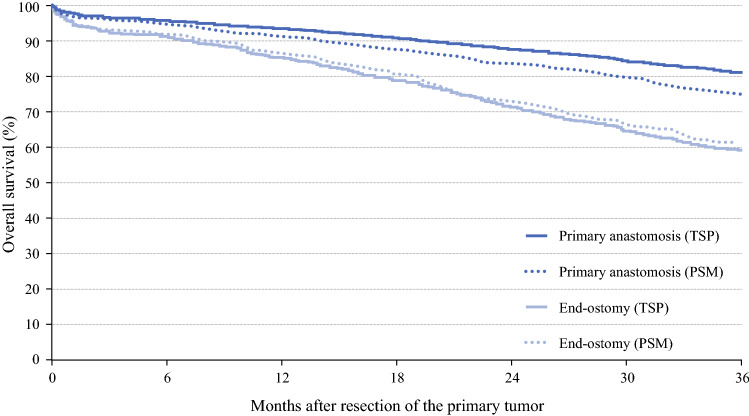
Three-year overall survival of the total study population and the propensity score matched sample according to surgical resection with primary anastomosis versus end-ostomy. *TSP* total study population, *PSM* propensity score matched sample

**Fig. 2.**
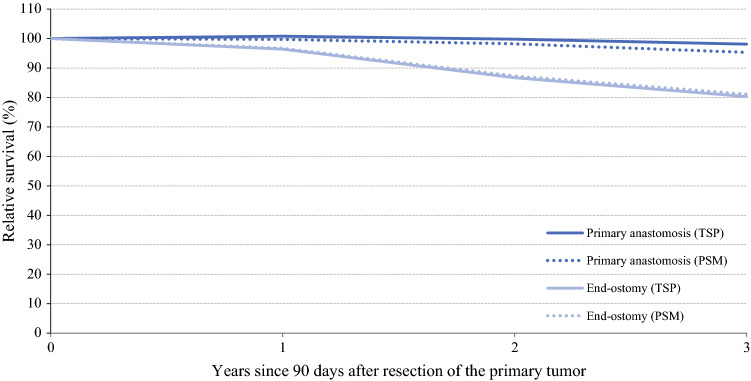
Three-year relative survival of the total study population and the propensity score matched sample according to surgical resection with primary anastomosis versus end-ostomy. *TSP* total study population, *PSM* propensity score matched sample The lines for end-ostomy (TSP) and end-ostomy (PSM) are overlapping

In the total study population, the median follow-up time was 37 months. Crude 3-year overall survival was 81.2% for patients in the PA group versus 58.7% in the EO group (*p* < 0.0001) (Fig. 1). Furthermore, in multivariable analysis, the risk of death was higher in patients with EO compared with those with PA (Table 4). Subgroup analyses among (1) patients with known comorbidity and (2) patients who did not receive (neo)adjuvant treatment did not change this (results not shown). Differences between the PA and EO groups were still prominent in relative survival, with significantly higher relative survival among patients in the PA group, both in univariable and multivariable analyses (Fig. 2, Table 4).

**Table 4.** Overall and relative survival and risks of death according to surgical resection with primary anastomosis versus end-ostomy

	OS		RS	
	Crude 3-year OS (%)	Adjusted HR of death^a^ (95% CI)	Crude 3-year RS (%)	RER of death^a^ (95% CI)
*Total study population*				
Primary anastomosis End-ostomy	81.2 58.7	1.00 (reference) **1.59 (1.35–1.87)**	98.1 80.3	1.00 (reference) **2.55 (1.64–3.97)**
*PSM sample*				
Primary anastomosis End-ostomy	75.1 61.5	1.00 (reference) **1.61 (1.32–1.97)**	95.3 81.1	1.00 (reference) **2.23 (1.34–3.72)**

*OS* overall survival, *HR* hazard ratio, *CI* confidence interval, *RS* relative survival, *RER* relative excess risk of death, *PSM* propensity score matched, *ASA* American Society of Anesthesiologists

^a^Adjusted for sex, age, ASA classification, performance status, year of diagnosis, tumor location, tumor stage, differentiation grade, resection type, surgical approach, prolonged hospital admission, neoadjuvant and adjuvant treatment

In the PSM sample, the median follow-up time was 35 months. Differences in crude 3-year overall and relative survival between patients in the PA group and patients who received an EO remained significant, also in multivariable analyses (Table 4).

## Discussion

To our knowledge, this is the first nationwide population-based study focused on the short-term outcomes and 3-year survival in elderly patients undergoing left-sided colon and proximal rectal cancer surgery with PSM. Our data suggest non-inferiority of PA compared to EO in mortality up to 90 days, analyzing PSM samples. Therefore, both patients and surgeons should evaluate the true benefits of an EO, based on alleged mortality risks, within the first month after surgery.

Postoperative hospital stay was longer and more often prolonged in EO patients, also after multivariable analysis and PSM. Pre-existent comorbidity or functional dependency may lead to an extended length of stay. Furthermore, increased length of stay in EO patients might be attributable to the fact that they are not independent in their stoma care when they are medically ready to be discharged. Arranging home-visiting nursing services often takes a couple of days.^11^ Worth noting is that an easy standardized in-hospital educational stoma pathway improves the level of independence in new stoma patients and reduces the need for home-visiting nursing services^12^ Pre- and postoperative stoma education has shown to be effective in reducing the postoperative hospital stay in a younger population.^13^ It is a challenge to achieve the same positive effects of stoma educational pathways in the elderly population, and this may be an interesting subject for future research.

Deviating ostomies do not seem to influence the incidence of an anastomotic leakage in PA patients in our study. These findings, as well as the fact that deviating ostomies seem to ameliorate the consequences of a leak, have been previously reported.^14^,^15^ Our registry did not encompass details such as postoperative reinterventions, intensive care unit (ICU) admissions, or readmissions after discharge; therefore, we cannot conclude that deviating ostomies reduce clinically relevant leakages or possible consequences of a leak in the Dutch elderly population. Furthermore, the fact that an anastomotic leakage is defined in many different ways, makes it hard to compare findings between studies. This database only registers anastomotic leakages based on radiological findings (as stated above), which might have resulted in an underrepresentation. However, the definition of anastomotic leakage for the NCR did not change over time, therefore any possible underrepresentation would be the same for the total study population.

A striking finding in our study is the difference in overall and relative long-term (3-year) survival in disadvantage of EO patients. The decreased overall and relative survival in EO compared with PA was significant in univariable, multivariable, and PSM analyses. Even though the impact of an ostomy on quality of life in elderly patients has been previously reported,^8^,^16^ little can be found on the impact of an ostomy on the survival of elderly patients after CRC surgery. Our data show that patients with two or more comorbidities are more likely to receive an EO; however, univariable and multivariable analysis show that this degree of comorbidity does not influence survival.

Studies have shown that postoperative ostomy-related complications such as prolapse, necrosis, stenosis, retraction, leakage, and others can be as high as up to 70%.^17^ The possible complications or reoperations may worsen quality of life, mental status, or social functioning and could be detrimental for the elderly patient. This effect may be an additional cause for the significantly higher 60- and 90-day postoperative mortality that we found in elderly EO patients. Multivariable analysis showed that age ≥85 years, ASA IV, perforation at the time of surgery, and open surgery are not only risk factors for receiving an EO but also for dying within 90 days postoperatively. Various factors that may or may not be obvious, detected at the initial outpatient assessment but not included in the database, might have led to the surgeons’ choice for an EO instead of an anastomosis. For example, the severity of comorbidities or the interplay between comorbidity and functional status might be such factors. Ultimately, no method reduces confounding by unmeasured variables. Indeed, frailty (vulnerability due to a decline of interrelated physiological systems), weight loss, and disability (presence of restriction in at least one activity of daily living) are a few examples of those factors that influence the vulnerability and survival of the elderly patient.^18^,^19^ Specific preoperative assessment, such as a comprehensive geriatric assessment (CGA) helps in predicting postoperative morbidity and mortality.^20^ In particular, dependency in instrumental activities of daily living, depression, polypharmacy, and impaired nutrition are important in predicting postoperative complications and early mortality.^20^–^23^ Previous research has shown that the occurrence of complications was the strongest risk factor for reduced survival in octogenarians.^24^ These results emphasize the importance of proper outpatient clinic consultation and the need for registration of the appropriate information regarding elderly patients beyond the standard given or measurable information that can be found in the medical charts. Performance scores, level of frailty, or CGA can be routinely assessed in clinical practice but unfortunately are not routinely documented in the charts or the data registries. In contrast, long-term survival is determined by a more complex interplay of non-surgical factors.

This study is also limited due to its retrospective, observational character and by the fact that occurrence of complications (other than anastomotic leakage and abscess) and causes of death are not registered in the NCR. The lack of complete information regarding the severity of comorbidities or performance scores are major limitations of this study, since both are important factors that can influence postoperative morbidity and survival. Relative survival was used in an effort to match cancer-specific survival as an estimation. This unfortunate shortcoming in the NCR data leaves many unknowns in our search for the exact causes of the survival differences in our elderly patients.

The downside of using PSM analysis is that the exclusion of patients from this analysis leads to loss of power. Nevertheless, PSM ensured the comparability of patients in both analyzed groups and provides additional information on subgroups in addition to the usual analysis in population-based data registries.

Relevant focus for further research would be to include more extensive data on performance scores, CGA, comorbidities, and postoperative complications that could lead to a European equivalent of the ACS NSQIP calculator and a better understanding of the survival and optimal treatment for our elderly patients with left-sided CRC. This information could be important for the decision making on surgical treatment in the elderly. Furthermore, repeating this analysis in 5 or 10 years, to evaluate the possible changes in EO rates and survival over time, as well as evaluation of interhospital variation in EO rates, would enrich the information for this decision making.

Despite the limitations of this study, one of its strengths is that it is based on the most comprehensive nationwide cancer registry with survival information that we have in The Netherlands. It shows real-life data and is a representation of our national elderly population with CRC. The discrepancies in 60- and 90-day mortality, as well as overall and relative survival, between patients with a PA and EO may be biased due to the different patient-specific factors, even though we have tried to correct for this using univariable, multivariable, and PSM analyses. Since there is significant difference in short-term mortality and overall and relative survival between patients with PA or EO in favor of PA, one might advocate that it is advisable to try to avoid the use of EO. A critical assessment on comorbidities, potential handling of an EO, age, and tumor stage will be necessary to argue in favor of an EO.

## Supplementary Information

Below is the link to the electronic supplementary material.

Supplementary file1 (DOCX 16 KB)
