# Supplementary material for: Primary Anastomosis Versus End-Ostomy in Left-Sided Colonic and Proximal Rectal Cancer Surgery in the Elderly Dutch Population: A Propensity Score Matched Analysis
Source: Ann Surg Oncol. 2021 Apr 25;28(12):7450–60. doi: 10.1245/s10434-021-09976-y (PMC8519826; doi:10.1245/s10434-021-09976-y)
Supplement: Supplementary file 1 — Supplementary file1 (DOCX 16 KB) [file 10434_2021_9976_MOESM1_ESM.docx]

**SUPPLEMENTARY TABLE S1** Crude percentages, univariable and multivariable odds ratios for postoperative 90-day mortality among the total study population and the propensity score matched sample

|  | Total study population | | | Propensity score matched sample | | |
| --- | --- | --- | --- | --- | --- | --- |
|  | **%** | Univariable OR (95% CI) | Multivariable OR* (95% CI) | % | Univariable OR (95% CI) | Multivariable OR* (95% CI) |
| **Sex**  Male  Female | 4.5%  3.7% | 1.00 (reference)  0.82 (0.58-1.17) | 1.00 (reference)  **0.64 (0.44-0.93)** | 4.6%  4.9% | 1.00 (reference)  1.07 (0.65-1.76) | 1.00 (reference)  0.95 (0.56-1.63) |
| **Age**  75-79 years  80-84 years  ≥85 years | 2.3%  4.6%  9.6% | 1.00 (reference)  **2.09 (1.36-3.20)**  **4.57 (2.97-7.03)** | 1.00 (reference)  **1.71 (1.10-2.67)**  **3.53 (2.21-5.65)** | 2.1%  5.1%  8.5% | 1.00 (reference)  **2.50 (1.23-5.08)**  **4.33 (2.12-8.85)** | 1.00 (reference)  **2.43 (1.16-5.07)**  **5.55 (2.58-11.92)** |
| **ASA classification**  I  II  III  IV | 0.6%  2.8%  5.8%  10.8% | 0.19 (0.03-1.37)  1.00 (reference)  **2.13 (1.46-3.10)**  **4.14 (1.80-9.53)** | 0.24 (0.03-1.78)  1.00 (reference)  **1.52 (1.02-2.27)**  **2.44 (1.01-5.90)** | 2.3%  3.0%  5.4%  15.0% | 0.77 (0.10-5.90)  1.00 (reference)  **1.86 (1.04-3.32)**  **5.71 (2.14-15.23)** | 1.24 (0.16-9.93)  1.00 (reference)  1.79 (0.97-3.30)  **6.22 (2.13-18.17)** |
| **Performance status**  0  1  2-4 | 2.3%  1.8%  7.6% | 1.00 (reference)  0.76 (0.33-1.73)  **3.51 (1.59-7.73)** | 1.00 (reference)  0.62 (0.27-1.43)  2.14 (0.93-4.90) | 2.4%  2.2%  5.9% | 1.00 (reference)  0.90 (0.25-3.25)  2.50 (0.69-9.13) | 1.00 (reference)  0.71 (0.19-2.65)  1.73 (0.44-6.83) |
| **Year of diagnosis**  2015  2016  2017 | 4.3%  4.0%  4.4% | 1.00 (reference)  0.92 (0.60-1.39)  1.01 (0.67-1.53) | 1.00 (reference)  0.91 (0.58-1.42)  1.07 (0.68-1.68) | 3.3%  5.1%  5.9% | 1.00 (reference)  1.54 (0.81-2.96)  1.82 (0.97-3.43) | 1.00 (reference)  1.69 (0.83-3.42)  1.94 (0.96-3.95) |
| **Location of the tumor**  Left-sided colon  Proximal rectum | 4.5%  3.3% | 1.00 (reference)  0.73 (0.46-1.15) | 1.00 (reference)  1.09 (0.57-2.06) | 4.9%  3.7% | 1.00 (reference)  0.74 (0.35-1.58) | 1.00 (reference)  1.47 (0.35-6.25) |
| **Tumor stage**  I  II  III | 2.9%  4.7%  4.6% | 1.00 (reference)  **1.67 (1.03-2.69)**  1.61 (0.99-2.63) | 1.00 (reference)  1.05 (0.63-1.75)  1.26 (0.74-2.13) | 4.1%  5.1%  4.6% | 1.00 (reference)  1.27 (0.63-2.55)  1.12 (0.54-2.34) | 1.00 (reference)  0.96 (0.46-2.03)  0.95 (0.43-2.11) |
| **Differentiation grade**  Well/moderate  Poor/undifferentiated | 4.2%  6.8% | 1.00 (reference)  1.66 (0.88-3.14) | 1.00 (reference)  1.27 (0.64-2.53) | 4.7%  6.3% | 1.00 (reference)  1.36 (0.53-3.50) | 1.00 (reference)  1.09 (0.39-3.08) |
| **Ileus**  No  Yes | 4.0%  7.6% | 1.00 (reference)  **1.99 (1.21-3.25)** | 1.00 (reference)  0.83 (0.47-1.46) | 4.6%  6.3% | 1.00 (reference)  1.40 (0.72-2.74) | 1.00 (reference)  0.89 (0.41-1.93) |
| **Perforation**  No  Yes | 4.0%  10.9% | 1.00 (reference)  **2.93 (2.53-5.62)** | 1.00 (reference)  **2.28 (1.12-4.66)** | 4.5%  11.1% | 1.00 (reference)  **2.63 (1.08-6.39)** | 1.00 (reference)  **3.09 (1.15-8.33)** |
| **Resection type**  Transversum resection  Left hemicolectomy  Sigmoid resection  Low anterior resection | 5.1%  6.2%  4.2%  2.6% | 0.82 (0.39-1.73)  1.00 (reference)  **0.66 (0.44-0.99)**  **0.40 (0.23-0.70)** | 0.73 (0.33-1.60)  1.00 (reference)  0.66 (0.42-1.03)  0.54 (0.27-1.10) | 3.2%  7.4%  4.3%  3.9% | 0.41 (0.09-1.82)  1.00 (reference)  **0.56 (0.32-0.99)**  0.50 (0.22-1.17) | 0.28 (0.06-1.34)  1.00 (reference)  0.73 (0.39-1.38)  0.61 (0.14-2.67) |
| **Surgical approach**  Laparoscopic  Laparoscopic with conversion to open  Open | 3.2%  4.6%  8.5% | 1.00 (reference)  1.48 (0.85-2.56)  **2.82 (1.93-4.12)** | 1.00 (reference)  1.07 (0.60-1.91)  **1.87 (1.20-2.92)** | 3.9%  5.0%  6.8% | 1.00 (reference)  1.30 (0.63-2.67)  **1.78 (1.03-3.09)** | 1.00 (reference)  1.14 (0.53-2.46)  **1.98 (1.03-3.79)** |
| **Neo-adjuvant treatment**  None  Radiotherapy  Chemoradiation | 4.4%  2.8%  2.1% | 1.00 (reference)  0.64 (0.26-1.57)  0.47 (0.12-1.94) | 1.00 (reference)  0.86 (0.31-2.43)  1.03 (0.23-4.69) | 4.9%  1.7%  3.3% | 1.00 (reference)  0.33 (0.05-2.40)  0.67 (0.09-4.97) | 1.00 (reference)  0.39 (0.04-3.36)  1.65 (0.18-15.09) |
| **Ostomy group**  Primary anastomosis  End-ostomy | 3.4%  7.7% | 1.00 (reference)  **2.38 (1.66-3.41)** | 1.00 (reference)  1.29 (0.84-1.99) | 4.0%  6.9% | 1.00 (reference)  **1.77 (1.05-2.96)** | 1.00 (reference)  **1.89 (1.10-3.26)** |

***Adjusted for all variables listed. ASA classification unknown, performance status unknown, differentiation grade unknown, ileus unknown and perforation unknown were included in the analysis but results not shown
